# A cross‐sectional study of the vulval dermatology clinic population over a 4‐year period and development of a national vulval disease database

**DOI:** 10.1002/ski2.446

**Published:** 2024-10-28

**Authors:** Marianne de Brito, Lisa Kirby, Kate Lomas, Aysha Javed, Caroline Owen, Rosalind Simpson

**Affiliations:** ^1^ Department of Dermatology Blackburn Hospital East Lancashire Hospitals NHS Trust Blackburn UK; ^2^ Centre of Cell Biology and Cutaneous Research Blizard Institute Queen Mary University of London London UK; ^3^ Centre of Evidence Based Dermatology School of Medicine University of Nottingham Nottingham UK; ^4^ School of Medicine University of Nottingham Nottingham UK; ^5^ The Countess of Chester Hospital NHS Foundation Trust Chester UK

## Abstract

**Background:**

Vulval disease significantly impacts quality of life but is historically under‐researched. The epidemiology and aetiology of many vulval conditions is unclear. Data to optimise patient care are lacking.

**Objectives:**

To describe the population attending a specialist vulval dermatology clinic and achieve consensus amongst vulval experts on data items to be collected for a future national vulval database.

**Methods:**

This descriptive cross‐sectional study analysed data that was prospectively collected during clinical contact with consecutive new patients at a vulval dermatology clinic over 4 years.

A two‐stage electronic‐Delphi survey was performed with British vulval experts. Consensus was defined as ≥75% agreement on items for inclusion.

**Results:**

The database included 424 (including 29 paediatric) patients. Most patients were White British (71%), with a significant Asian population (13%). Long symptom duration (9.5% > 10 years) and multiple diagnoses, up to 4, were common. Exploratory associations were identified between irritant contact dermatitis and urinary and faecal incontinence, frequent vulval washing and lichen simplex, urinary incontinence and lichen sclerosus and a negative association between candidiasis and age.

Following two rounds of the electronic‐Delphi survey, consensus was achieved for 18 items that 28 participants agreed were important for a future database.

**Conclusions:**

We report disease incidence, patient pathways, outcome measures and potential associations. Though not generalisable, this large UK‐based study could inform future projects to improve patient care and support ongoing research, such as a national vulval disease database, for which we also achieve expert consensus on the most valuable items to include.

**What is already known?**

- Vulval disease has a profound impact on sexual functioning, self‐image and relationships but is historically under‐researched. The epidemiology and aetiology of many vulval conditions are unclear and data to optimise patient care, patient outcomes and service design are lacking. Prospective disease databases can be a valuable resource to improve patient care and support the development of research aims.

**What does this study add?**

- This prospective cross‐sectional study, using data collected during routine clinical practice, describes the patient population attending a specialist vulval dermatology clinic to give insights on disease incidence, associations and patient pathways. We also report expert agreement, via a two‐stage Delphi study, on the most valuable items to include in a future national vulval disease database. A future clinical database could improve patient care and support research into this neglected area.

## 1 INTRODUCTION

Vulval clinics provide specialist care for women with vulval disease, which is frequently diagnostically challenging. Specific conditions can affect vulval skin, such as lichen sclerosus, and common dermatoses may present very differently in vulval skin compared to elsewhere. Furthermore, management presents unique difficulties as vulval skin is easily irritated by friction, heat, sanitary products, discharge and changes in flora. Exposure to moisture from sweat, urine and faeces can irritate and trigger flares, with delayed response to treatment.^1^

Vulval disease is common and causes considerable impact on quality of life and function. Vulval skin conditions can be perceived as embarrassing and can interfere with sexual functioning, self‐image and interpersonal relationships.^2^ Disease burden in atopic dermatitis and psoriasis is increased when the genital skin is involved.^2^, ^3^, ^4^

Despite this, vulval disease is historically under‐researched. Genital disorders are under‐represented in high‐impact dermatology journals^3^ and vulval research quality is low, comprising mainly case reports and series.^5^ Furthermore, LS is recognized by the European Commission as a high‐burden under‐researched medical condition.^6^ Little is known of the epidemiology and aetiology of lichen sclerosus, such as many other vulval conditions.^7^ Optimising patient care, outcomes and service design depends upon robust data which is currently lacking. Patient registries and databases are valuable and widely used in dermatology for study of rare diseases and post‐surveillance drug monitoring of common diseases, but none yet exist for vulval disease.^8^

This study has two aims: first, to describe the patient population attending a specialist vulval dermatology clinic between 2017 and 2020 using prospectively collected data entered during routine clinical practice; second, to explore clinicians views on design of a potential future national vulval disease database, using an electronic‐Delphi technique.

## 2 METHODS

### 2.1 Cross‐sectional study

The cross‐sectional study utilised data collected during routine clinical practice, prospectively entered into the East Lancashire Hospitals NHS Trust vulval dermatology database (2017–2020). Anonymised data were collected and entered in a Microsoft Access database hosted securely by the East Lancashire Teaching Hospitals for consecutive new adult and paediatric patients attending a specialist vulval dermatology clinic. There were no exclusions. All patients were assessed by a consultant dermatologist with a specialist interest in vulval disease. Diagnoses were made predominantly on clinical grounds, but where the clinical presentation was atypical, or there were concerns about malignancy, biopsy was undertaken.

Data collected included demographics, BMI, parity, ethnicity, referral source, symptom duration, diagnosis, patient‐reported incontinence (faecal, urinary), vulval hygiene practices and outcome measures at presentation: dermatology life quality index (DLQI), visual analogue score, international consultation on incontinence questionnaire—urinary incontinence short form (ICIQ) score and sexual function. The protocol for this study was prospectively published on the Centre of Evidence Based Dermatology's protocol portal.^9^

### 2.2 Statistical methods

The database was interrogated at East Lancashire Hospitals Trust on 9th January 2020. A data quality check performed separately confirmed all data were extracted correctly. Following advice from the University of Nottingham Research Governance Office, ethical approval was not required as data were anonymised and unlinked throughout data analysis. The data custodian at the collection site granted permission to use these data in the way described. A subset of this database has been previously reported on.^1^ Statistical analysis was performed using Stata version 17.0. Regression analysis was exploratory and hence uncorrected. Missing data were excluded from analysis.

### 2.3 Delphi study

A two‐stage electronic‐Delphi survey, led by the University of Nottingham (KL), was undertaken. Experts were identified and invited to participate via email, through the British Society for the Study of Vulval Disease (BSSVD) membership. The protocol was prospectively published.^10^

Using Microsoft Forms, experts rated the importance of a list of items collected during routine clinics for their inclusion in a national database, as ‘not important’, ‘important but not critical’, ‘critical’ or ‘unable to comment’. Participants were able to comment on the listed items and enter additional items they felt were missing from the original list. Suggestions from participants were thematically analysed to produce new items. Comments on existing items were considered and wording changes implemented where relevant. The process was conducted anonymously. Round 1 results, as well as new participant‐suggested items and re‐worded items, were presented in round 2 for further rating. Considering the group scores from round 1, participants were able to change their scores in round 2. Consensus was defined a priori as ≥75% agreement on item importance. Items reaching consensus as ‘critical’ should be strongly considered for inclusion in a future database.

## 3 RESULTS

### 3.1 Cross‐sectional study

Data were collected from 423 patients, including 29 paediatric patients. Demographics are shown in Table 1. Median age was 55 years old (range: 3–90). Median BMI for patients ≥21 years old was 27 (IQR 24–32), calculated for 358/381. Median parity for adult patients (≥16 years old) was 2 (IQR 1–3, 376/392). The most common ethnic group was White British (307/420, 73.1%), with a significant Asian population (55/420, 13.1%) of which 8.1% were Pakistani or British Pakistani.

**TABLE 1:** Patient characteristics of included patients including demographics, referral source and duration of symptoms at time of presentation.

		Paediatric (<16 years old)	Adult (16+ years old)	Total
Number of patients		29	394	423
Median age (range)		55 (3–90)^a^		
Ethnicity	White British	20/28 (71.4%)	287/392 (73.2%)	307/420 (73.1%)
	Not stated	1/28 (3.57%)	38/392 (9.64%)	39/420 (9.28%)
	Pakistani or British Pakistani	3/28 (10.7%)	31/392 (7.91%)	34/420 (8.09%)
	Indian or British Indian	0/28 (0.00%)	15/392 (3.82%)	15/420 (3.57%)
	Other White	1/28 (3.57%)	9/392 (2.30%)	10/420 (2.38%)
	Other Asian or other British Asian	2/28 (7.14%)	3/392 (0.01%)	5/420 (1.19%)
	Mixed (any)	0/28 (0.00%)	4/392 (1.02%)	4/420 (0.95%)
	Any other ethnic group	0/28 (0.00%)	3/392 (0.01%)	3/420 (0.01%)
	White Irish	0/28 (0.00%)	1/392 (0.003%)	1/420 (0.002%)
	Bangladeshi or British Bangladeshi	1/28 (3.57%)	0/392 (0.00%)	1/420 (0.002%)
	African	0/28 (0.00%)	1/392 (0.003%)	1/420 (0.002%)
Referral source	General practice	10 (33.3%)	174 (45.6%)	184 (43.8%)
	Gynaecology	10 (33.3%)	115 (30.1%)	125 (29.8%)
	Dermatology	6 (20.0%)	33 (8.64%)	39 (9.29%)
	Urogynaecology	‐	14 (3.66%)	14 (3.33%)
	GP with special interest in dermatology	2 (6.67%)	10 (2.62%)	12 (2.86%)
	Physiotherapy	‐	10 (2.62%)	10 (2.38%)
	Psychosexual	‐	4 (1.05%)	4 (0.95%)
	Genito‐urinary medicine	‐	3 (0.79%)	3 (0.71%)
	Other	2 (6.67%)	19 (4.97%)	21 (5.00%)
Duration of symptoms at presentation	0–6 months	2 (6.45%)	23 (5.91%)	25 (5.95%)
	6–12 months	9 (29.0%)	119 (30.6%)	128 (30.5%)
	1–3 years	10 (23.3%)	107 (27.5%)	117 (27.9%)
	3–5 years	7 (22.6%)	51 (13.1%)	58 (13.8%)
	5–10 years	3 (9.68%)	50 (12.9%)	53 (12.6%)
	10+ years	‐	39 (10.0%)	39 (9.29%)

^a^ Missing data for age: 3.

Referral source is shown in Table 1. Most patients were referred from general practice (GP, 44.6%), but others were referred from gynaecology (30.6%), dermatology (9.4%), urogynaecology (3.37%), GP with special interest in dermatology (2.89%), physiotherapy (2.41%), psychosexual (0.96%) and genito‐urinary medicine (0.72%).

The median symptom duration (Table 1) at first presentation to vulval clinic was 1–3 years, although 58/420 (13.8%) had had symptoms for 3–5 years, 53/420 (12.6%) for 5–10 years and 40/420 (9.52%) had had symptoms for more than 10 years.

Clinical diagnosis is shown in Table 2. Most patients had one diagnosis, 297/421 (70.6%). However, 97/421 (23.0%) had two diagnoses, 22/421 (5.23%) had three diagnoses and 4/421 (0.95%) had four diagnoses. The most common primary diagnosis was lichen sclerosus (137/421 32.5%); the next most common were atopic eczema (75/421, 17.8%), vulvodynia (29/421, 6.89%), irritant contact dermatitis (ICD) (27/421, 6.41%) candidiasis (23/421, 5.46%), lichen simplex (20/421, 4.75%) and allergic contact dermatitis (11/421, 2.61%). There were, in total, more than 40 different diagnoses.

**TABLE 2:** Clinical diagnoses of included patients.

	Number of patients (%)
Number of diagnoses	
0	1
1	297
2	97
3	22
4	4
Primary diagnosis	
Lichen sclerosus	137
Atopic eczema	75
Other	31
Lichen planus	29
Vulvodynia	29
Irritant contact dermatitis	27
Candidiasis	23
Lichen simplex	20
Allergic contact dermatitis	11
Lesion	11

*Note*: Number of diagnoses and number of patients with each diagnosis. Some patients had several different diagnoses.

Table 3 shows urinary, faecal and sexual function and hygiene practices. Urinary incontinence was reported by 173/415 (41.7%), whilst 26/398 (6.5%) reported faecal incontinence. Vulval washing more than once daily was reported in 98/407 (24.1%) and 8/407 (2.0%) reported vulval washing 10+ times/day. Pre‐prayer washing was practised by 42/423 (9.9%) and pubic hair was removed in 181/423 (42.8%). Of those sexually active, most (218/381, 87.2%) found sexual activity problematic. Median DLQI at presentation was 9 (IQR 4–13).

**TABLE 3:** Urinary, faecal and sexual function, and hygiene practices.

		Paediatric (<16 years old)	Adult (16+ years old)	Overall
Urinary incontinence	Yes	7 (23.3%)	166 (43.1%)	173/415 (41.7%)
Faecal incontinence	Yes	1 (3.85%)	25 (6.72%)	26/398 (6.5%)
Pre‐prayer washing	Yes	5 (16.1%)	37 (9.44%)	42/423 (9.9%)
Sexual function	Not relevant	Not applicable/not asked	163/413 (39.5%)	
	No problems		32/413 (7.7%)	
	Possible, but uncomfortable		134/413 (32.4%)	
	Impossible		84/413 (20.3%)	
Pubic hair removal	Yes	Not applicable/not asked	181/423 (42.8%)	
Vulval washing times per day	<1	9/407 (2.2%)		
	1	300/407 (73.7%)		
	2	43/407 (10.6%)		
	3	9/407 (2.2%)		
	4	9/407 (2.2%)		
	5	15/407 (3.7%)		
	6	10/407 (2.5%)		
	7	0/407 (0%)		
	8	4/407 (1.0%)		
	9	0/407 (0%)		
	10+	8/407 (2.0%)		

Vulval biopsy occurred in 148/420 (35.2%) patients, prior to or following referral to the specialist vulval service. The biopsy was concordant with clinical diagnosis in 129/148 (87.2%) patients. Patch testing was recommended for 58/415 (14.0%) patients. Following patch testing (*n* = 49, Table 4), the most common allergens were nickel (15/49), methylisothiazolinone (10/49), cobalt (8/49) and methylchloroisothiazolinone (8/49).

**TABLE 4:** Most frequent contact allergens in patients having undergone patch testing (*n* = 49).

Contact allergen	Number of patients
Nickel	15
Cobalt	9
Methylisothiazolinone (MI)	9
Methylchloroisothiazolinone (MCI)	7
Balsam of Peru	4
Sodium metabisulphite	4
Paraphenylenediamine	3
Negative	2
Lignocaine	2

*Note*: List of positive contact allergens affecting 1 person (not shown in table): Oak moss, Framycetin, Colophony, Epoxy resins, Fragrance, Geraniol, Methyldibromoglutaronitrile, Quaternium 15, Parabens, Toluenesulphonamide, Red phosphorous, Amino‐azabenzene, Thiuram mix, Potassium dichromate, Amycinnemaldehyde, Fragrance mix, Inconclusive, Clioquinol, Quinoline mix, Sorbic acid, Coal tar, p‐chloro‐m‐cresol, Disperse orange, Pivalone, Thiomersal, Formaldeyde resin, DMD Hydantoin, Methacrylates, Drapolene.

Logistic regression analysis between washing frequency, urinary and faecal incontinence and pre‐prayer washing with primary diagnoses revealed the associations shown in Table 5. Washing frequency was associated with lichen simplex (*p* < 0.001, odds ratio (OR) 1.43) and eczema (*p* = 0.002, OR 1.20). UI was positively associated with LS (*p* < 0.00.1, OR 2.86) and ICD (*p* < 0.001, OR 4.95) and negatively associated with candidiasis (*p* < 0.001, OR 0.29). Faecal incontinence was associated with LS/LP overlap (*p* = 0.005, OR 7.96) and ICD (*p* < 0.001, OR 7.13). Pre‐prayer washing was associated with lichen simplex (*p* < 0.001, OR 9.60).

**TABLE 5:** Associations between hygiene practices and incontinence and vulval conditions.

	Association	Odds ratio	*p* value
Washing frequency	Lichen simplex	1.43	<0.001
	Eczema	1.20	0.002
Urinary incontinence	Lichen sclerosus	2.86	<0.001
	Irritant contact dermatitis	4.95	<0.001
	Candidiasis	0.29	<0.001
Faecal incontinence	LS/LP overlap	7.96	0.005
	Irritant contact dermatitis	7.13	<0.001
Pre‐prayer washing	Lichen simplex	9.60	<0.001

### 3.2 Delphi survey

Regarding the Delphi survey (Table 6), round one comprised 46 participants, representing 6 specialties: dermatology, gynaecology, sexual health, nursing, GP and gynae–oncology. 47 data items were surveyed, with 10/47 reaching consensus as ‘critical’ and removed from inclusion in round 2. The 37 remaining database items were carried forward and 26 new items were added into the survey. Round 2 was completed by 28 participants. Following both rounds, consensus was achieved for 18 database headings.

**TABLE 6:** Most valuable data items to be included in a national vulval disease database as ascertained by 2‐round Delphi study of British vulval experts where consensus was defined as 75%+ agreement on a data item being critical for inclusion.

	Item
1	Age
2	Presenting symptoms
3	Symptom duration
4	Washing method including use of potential irritants
5	Previous biopsy
6	Biopsy result
7	Primary diagnosis
8	Treatment given
9	Treatment response
10	Adverse treatment effects
11	Dermatological history
12	Systemic diseases
13	Malignancy/cervical intraepithelial neoplasia
14	Menopausal status
15	Urinary incontinence
16	Additional diagnosis
17	Previous vulval diagnoses
18	Treatment(s) prior to referral

## 4 DISCUSSION

Notable findings of our descriptive study of vulval clinic patients are the long median symptom duration at first presentation of 1–3 years, though 10% of adult patients reported symptoms for >10 years. The complexity of presentations is demonstrated by 29% of patients having multiple vulval diagnoses recorded. We also found a significant impact on quality of life, with a median DLQI at first presentation of 9, with frequent impact on sexual function; of those sexually active, 34% reported sexual activity was impossible. This demonstrates clinical need for a specialist vulval clinic with networks to enable referral of patients to key services such as specialised physiotherapy, psychosexual therapy, continence advisory services and pain management as patients need a dedicated service to address their needs comprehensively and holistically.^11^

Compared to previous UK‐based studies of vulval clinic patients, this is the largest and only prospective study. Wolpert at al showed a similarly long median symptom duration at first presentation (28.9 months) in 1999.^12^ This could be due to self ‐treatment, or disease treatment/control in GP/non‐superspecialist setting prior to referral, but the paucity and variable standard of UK vulval clinics is likely to have a role.^13^ The three other previous studies were concordant with ours regarding most common referral source (GP) and most frequent diagnoses: lichen sclerosus, eczema, vulvodynia, lichen planus and lichen simplex.^14^, ^15^, ^16^ Wolpert et al's most frequent diagnoses were contact dermatitis, vulvar vestibulitis and vulvodynia, possibly due to the different clinic population in genito‐urinary medicine.

Vulval clinic studies internationally also report lengthy waits and lichen sclerosus is frequently the most frequent diagnosis.^17^, ^18^, ^19^, ^20^ Conversely, a Swedish study reported localised provoked vulvodynia as the most frequent diagnosis, attributed to the relatively young population, whilst a German study reported vulvitis as the most common diagnosis, having grouped the different dermatitis' together.^21^, ^22^ The latter also confirmed significant impact on quality of life, (DLQI >10 in 55/140). Regarding sexual function, dyspareunia was frequently reported (67.6%)^21^ and 64% of women in a Brazilian study reported sexual inactivity, though causation by vulval disease was not ascertained.^19^

A recent systematic review of patch test‐identified allergens in vulval patients (including a large UK‐based study) was concordant with our findings, reporting nickel/cobalt as the most common, though of uncertain significance, alongside Balsam of Peru, MI/CMI and topical anaesthetic, which are more likely clinically relevant given these products may be exposed to the vulva.^23^, ^24^ However, we found no allergies to antibiotics and few to topical steroids, possibly because of the small numbers patch‐tested.

Associations were identified through exploratory regression analysis which has not undergone Bonferroni correction or correction for confounders. We replicate the association previously found in this database between urinary incontinence and lichen sclerosus.^1^ ICD was also associated with urinary incontinence. Candidiasis was negatively associated—we hypothesise this could be due to confounding related to age; candidiasis is less frequent in post‐menopausal adults who are also more likely to experience urinary incontinence.^1^, ^25^ We also found associations with faecal incontinence and ICD, hypothetically for the same reason as its association with urinary incontinence, and with LS/LP overlap—this is of uncertain clinical significance given the LS/LP group (those with evolving clinical or histological features between lichen sclerosus and lichen planus) was very small.

Pre‐prayer washing was reported by 10% of patients. This practice, observed in Islam, involves genital area washing pre‐prayer after certain circumstances such as post coitus, genital stimulation or touching that would leave a discharge, or use of the toilet immediately preceding prayers. In practice, washing may be performed more frequently than this, as highlighted in this study. Washing of the genitals is expected in Islam after each use of the toilet and must be done using water if available as toilet paper is not deemed sufficient to cleanse the genital skin. Additionally, Islam stipulates the removal of pubic hair for all followers to optimise hygiene levels. These practices may affect the skin barrier, irritating sensitive vulval skin, leading to diagnoses such as lichen simplex and ICD. Here, we found that pre‐prayer washing and frequent vulval washing were significantly associated with lichen simplex, which has not been previously reported or demonstrated in the literature. This shows the importance of awareness of and sensitively enquiring about vulval cultural practices, as there may be clinical implications. This should be an area for future study.

The limitations of this study are that it may not be representative of the UK population at large, describing a predominantly White population, with a significant minority of Asian, particularly Pakistani, ancestry. However, it shows the feasibility of data collection alongside routine clinical practice. Furthermore, via the electronic‐Delphi survey, we achieve consensus amongst a group of UK vulval dermatology experts regarding what data should be routinely collected to develop a national vulval disease registry. If this initiative was to proceed, the possibilities for collecting data to inform on epidemiology, service planning and treatment pathways would be significant. It could also be an invaluable resource for patient recruitment for clinical trials into historically under‐researched vulval disease.

At present, no vulval disease databases exist internationally, there are only cancer databases that include female genital cancers such as the UK National Cancer Data Repository and the American National Cancer Database—the latter includes information about treatments and outcomes, which has enabled research to show improved survival with definitive chemoradiotherapy rather than radiotherapy alone, which has advanced patient care.^26^, ^27^ This demonstrates the possible benefits of a database for patients with other vulval diseases.

## 5 CONCLUSION

This cross‐sectional study demonstrates the breadth, multiple‐diagnoses and impact of vulval disease in patients attending a vulval clinic, strengthening the need for specialist clinics. Though not generalisable, it could inform future projects such as a national vulval disease database to support patient care and research. We investigate the practicalities of such a database by carrying out an electronic‐Delphi survey to vulval experts, achieving consensus on 18 items for inclusion.

## CONFLICT OF INTEREST STATEMENT

None to declare.

## AUTHOR CONTRIBUTIONS

**Marianne de Brito**: Conceptualization (supporting); data curation (equal); formal analysis (lead); investigation (equal); methodology (equal); project administration (equal); software (equal); writing—original draft (lead); writing—review & editing (equal). **Lisa Kirby**: Conceptualization (equal); data curation (equal); formal analysis (supporting); investigation (equal); methodology (equal); project administration (equal); supervision (equal); writing—review & editing (equal). **Kate Lomas**: Conceptualization (equal); data curation (equal); formal analysis (equal); investigation (equal); methodology (equal); writing—original draft (equal); writing—review & editing (equal). **Aysha Javed**: Conceptualization (equal); data curation (equal); investigation (equal); methodology (equal); supervision (equal); writing—review & editing (equal). **Caroline Owen**: Conceptualization (lead); data curation (lead); formal analysis (equal); investigation (lead); methodology (equal); supervision (lead); writing—original draft (equal); writing—review & editing (equal). **Rosalind Simpson**: Conceptualization (lead); data curation (lead); formal analysis (equal); investigation (equal); methodology (equal); supervision (lead); writing—review & editing (lead).

## ETHICS STATEMENT

As data were anonymised and unlinked during processing and analysis, ethical approval was not required for this study. The data custodian at the East Lancashire Hospitals NHS Trust (the data collection site) provided the necessary permissions to use these data in the way described.

## PATIENT CONSENT

Not applicable.

## Supporting information

Supporting Information S1

## Data Availability

The data that support the findings of this study are available on request from the corresponding author. The data are not publicly available due to privacy or ethical restrictions.
