# Supplementary material for: A cross‐sectional study of the vulval dermatology clinic population over a 4‐year period and development of a national vulval disease database
Source: Skin Health Dis. 2024 Oct 28;4(6):e446. doi: 10.1002/ski2.446 (PMC11608872; doi:10.1002/ski2.446)
Supplement: Supplementary file 1 — Supporting Information S1 [file SKI2-4-e446-s001.docx]

**Supplementary information**

**Supplementary Table 1:**

Results of Round one Delphi survey. Items reaching 75% consensus as ‘critical’ in bold.

| Proforma item | Responses, n (%) |
| --- | --- |
|  | Critical Important Not Unable to  (not critical) important comment |
| Referral information | |

| Source of referral Primary/secondary/ tertiary | 9 (20) | 31 (67) | 6 (13) | 0 (0) |
| --- | --- | --- | --- | --- |
| Patient demographics |  |  |  |  |
| **Age** | **35 (76)** | 9 (20) | 1 (2) | 1 (2) |
| Ethnicity | 16 (35) | 26 (57) | 3 (7) | 1 (2) |
| BMI | 15 (33) | 26 (57) | 4 (9) | 1 (2) |
| History of presenting complaint |  |  |  |  |
| Date of first assessment | 29 (63) | 17 (37) | 0 (0) | 0 (0) |
| **Presenting symptoms e.g. pruritis, dyspareunia (free text)** | **41 (89)** | 5 (11) | 0 (0) | 0 (0) |
| **Duration of symptoms** | **41 (90)** | 5 (11) | 0 (0) | 0 (0) |
| History detail | 32 (70) | 13 (28) | 0 (0) | 1 (2) |
| Outcome measure scores |  |  |  |  |
| Dermatology Life Quality Index (DLQI) | 6 (13) | 24 (52) | 4 (9) | 12 (26) |
| Vulvar Quality of Life Index (VQLI) | 13 (28) | 15 (33) | 3 (7) | 15 (33) |
| Visual Analogue Score (VAS) of symptoms | 8 (17) | 26 (57) | 3 (7) | 9 (20) |
| International Consultation on Incontinence Questionnaire (ICIQ) | 4 (9) | 15 (33) | 9 (20) | 19 (39) |
| Hospital Anxiety and Depression Scale (HADS) | 3 (7) | 26 (57) | 8 (17) | 9 (20) |
| PHQ-9 (depression scale) | 1 (2) | 22 (48) | 11 (24) | 12 (26) |
| Outcome measure scores at presentation | 10 (22) | 22 (48) | 4 (9) | 10 (22) |
| Outcome measure scores at review | 10 (22) | 24 (52) | 3 (7) | 9 (20) |

| Outcome measure scores at discharge | 11 (24) | 24 (52) | 3 (7) | 8 (17) |
| --- | --- | --- | --- | --- |
| Past medical history |  |  |  |  |
| Past medical history atopy/psoriasis/malignancy/  autoimmune | 26 (63) | 17 (37) | 0 (0) | 0 (0) |
| Relationship/sexual function |  |  |  |  |
| Relationship status | 20 (44) | 24 (52) | 2 (4) | 0 (0) |
| Sexual activity at presentation/discharge | 28 (61) | 17 (37) | 0 (0) | 1 (2) |
| Gynae history |  |  |  |  |
| Gravida | 9 (20) | 28 (60) | 9 (20) | 0 (0) |
| Parity | 10 (22) | 27 (59) | 9 (20) | 0 (0) |
| Episiotomy or tear | 10 (22) | 31 (67) | 5 (11) | 0 (0) |
| Smear test history | 26 (57) | 19 (41) | 1 (2) | 0 (0) |
| Menopausal status | 34 (74) | 12 (26) | 0 (0) | 0 (0) |
| Contraception usage | 19 (41) | 21 (46) | 6 (13) | 0 (0) |
| Contraception method | 20 (44) | 21 (46) | 5 (11) | 0 (0) |
| Incontinence history |  |  |  |  |
| Urinary incontinence | 33 (72) | 13 (28) | 0 (0) | 0 (0) |
| Faecal incontinence | 27 (59) | 17 (37) | 1 (2) | 1 (2) |
| Use of incontinence pads | 33 (72) | 12 (26) | 0 (0) | 1 (2) |
| Family history |  |  |  |  |
| Family history atopy/psoriasis/  malignancy/autoimmune | 19 (41) | 26 (57) | 1 (2) | 0 (0) |
| Vulval hygiene |  |  |  |  |
| Washing frequency | 26 (57) | 19 (41) | 1 (2) | 0 (0) |
| **Washing method (e.g. wipes, soaps)** | **35 (76)** | 10 (22) | 1 (2) | 0 (0) |
| Pre- prayer washing | 15 (33) | 19 (41) | 4 (9) | 8 (17) |
| Hair removal | 18 (39) | 23 (50) | 4 (9) | 1 (2) |
| Hair removal method | 19 (41) | 22 (48) | 4 (9) | 1 (2) |
| Investigations |  |  |  |  |
| **Previous biopsy** | **37 (80)** | 9 (20) | 0 (0) | 0 (0) |
| Biopsy this episode | 34 (74) | 12 (26) | 0 (0) | 0 (0) |
| **Biopsy result** | **44 (96)** | 2 (4) | 0 (0) | 0 (0) |
| Patch test/patch test results | 26 (63) | 14 (30) | 0 (0) | 3 (7) |

| Swab/swab result | 31 (67) | 14 (30) | 0 (0) | 1 (2) |
| --- | --- | --- | --- | --- |
| Medical photography | 19 (41) | 22 (48) | 3 (7) | 2 (4) |
| Diagnosis |  |  |  |  |
| **Diagnosis** | **42 (91)** | 4 (9) | 0 (0) | 0 (0) |
| Treatments |  |  |  |  |
| Treatment(s) prior to referral | 34 (74) | 12 (26) | 0 (0) | 0 (0) |
| **Treatment given at outpatient appointment** | **43 (94)** | 3 (7) | 0 (0) | 0 (0) |
| Date treatment started/date treatment stopped | 31 (67) | 15 (33) | 0 (0) | 0 (0) |
| **Treatment response** | **44 (96)** | 2 (4) | 0 (0) | 0 (0) |
| **Adverse effects of treatment** | **42 (91)** | 4 (9) | 0 (0) | 0 (0) |

**Supplementary Table 2:**

Results of Round two Delphi survey. Items reaching 75% consensus as ‘critical’ in bold.

| Proforma Item | | Responses, n (%) | | | |
| --- | --- | --- | --- | --- | --- |
|  |  | Critical Important Not Unable to  (not critical) important comment | | | |
| Referral information | | | | | |
| Source of referral Primary/secondary/ | 1 (4) | | 26 (93) | 1 (4) | 0 (0) |
| tertiary  Specialty of referral | 1 (4) | | 25 (89) | 2 (7) | 0 (0) |
| 2 Week wait or referral | 14 (50) | | 11 (39) | 1 (4) | 2 (7) |
| Patient demographics |  | |  |  |  |
| Ethnicity | 13 (46) | | 13 (46) | 2 (7) | 0 (0) |
| BMI | 10 (36) | | 16 (57) | 2 (7) | 0 (0) |
| History of presenting complaint |  | |  |  |  |
| Date of first assessment | 18 (64) | | 9 (32) | 1 (4) | 0 (0) |
| History detail e.g. exacerbating and relieving factors | 16 (57) | | 12 (43) | 0 (0) | 0 (0) |

| Outcome measure scores |  |  |  |  |
| --- | --- | --- | --- | --- |
| Dermatology Life Quality Index (DLQI) | 3 (11) | 19 (68) | 2 (7) | 4 (14) |
| Vulvar Quality of Life Index (VQLI) | 8 (29) | 12 (43) | 1 (4) | 7 (25) |
| Visual Analogue Score (VAS) of symptoms | 6 (21) | 14 (50) | 2 (7) | 6 (21) |
| International Consultation on Incontinence Questionnaire (ICIQ) | 2 (7) | 14 (50) | 5 (18) | 7 (25) |
| Hospital Anxiety and Depression Scale (HADS) | 1 (4) | 22 (79) | 1 (4) | 4 (14) |
| PHQ-9 (depression scale) | 0 (0) | 20 (71) | 2 (7) | 6 (21) |
| Outcome measure scores at presentation | 7 (25) | 19 (68) | 1 (4) | 1 (4) |
| Outcome measure scores at review | 9 (32) | 18 (64) | 1 (4) | 0 (0) |
| Outcome measure scores at discharge | 12 (43) | 15 (54) | 1 (4) | 0 (0) |
| Past medical history |  |  |  |  |
| Past medical history | 18 (64) | 10 (36) | 0 (0) | 0 (0) |
| **Dermatological history e.g. atopy/psoriasis** | **21 (75)** | 7 (25) | 0 (0) | 0 (0) |
| **Systemic diseases e.g. autoimmunity** | **21 (75)** | 7 (25) | 0 (0) | 0 (0) |
| **Malignancy/cervical intraepithelial neoplasia** | **23 (82)** | 3 (11) | 0 (0) | 2 (7) |
| HPV vaccination status | 10 (36) | 16 (57) | 2 (7) | 0 (0) |
| HIV status | 8 (29) | 19 (68) | 1 (4) | 0 (0) |
| Diabetic status | 18 (64) | 10 (36) | 0 (0) | 0 (0) |
| Relationship/sexual function |  |  |  |  |
| Relationship status | 9 (32) | 18 (64) | 1 (4) | 0 (0) |
| Sexual activity at presentation/discharge | 18 (64) | 10 (36) | 0 (0) | 0 (0) |
| History of sexual dysfunction/ psychosexual problems Y/N | 18 (64) | 10 (36) | 0 (0) | 0 (0) |
| Gynae history |  |  |  |  |
| Gravida | 5 (18) | 18 (64) | 5 (18) | 0 (0) |
| Parity | 5 (18) | 19 (68) | 4 (14) | 0 (0) |
| Episiotomy or tear | 6 (21) | 19 (68) | 3 (11) | 0 (0) |
| Smear test history | 14 (50) | 13 (46) | 1 (4) | 0 (0) |
| **Menopausal status** | **21 (75)** | 7 (25) | 0 (0) | 0 (0) |
| Contraception usage | 9 (32) | 18 (64) | 1 (4) | 0 (0) |
| Contraception method | 11 (39) | 17 (61) | 0 (0) | 0 (0) |
| Method of delivery | 7 (25) | 19 (68) | 2 (7) | 0 (0) |
| HRT usage | 15 (54) | 13 (46) | 0 (0) | 0 (0) |
| Previous gynae infection e.g. thrush | 12 (43) | 16 (57) | 0 (0) | 0 (0) |

| Previous gynae surgery | 15 (54) | 12 (43) | 1 (4) | 0 (0) |
| --- | --- | --- | --- | --- |
| Incontinence and urological history |  |  |  |  |
| **Urinary incontinence** | **21 (75)** | 7 (25) | 0 (0) | 0 (0) |
| Faecal incontinence | 18 (64) | 9 (32) | 1 (4) | 0 (0) |
| Use of incontinence pads | 20 (71) | 8 (29) | 0 (0) | 0 (0) |
| Medical treatments for incontinence | 11 (39) | 16 (57) | 1 (4) | 0 (0) |
| Surgical treatment for incontinence | 14 (50) | 14 (50) | 0 (0) | 0 (0) |
| Family history |  |  |  |  |
| Family history atopy/psoriasis/malignancy/  autoimmunity | 8 (29) | 19 (68) | 1 (4) | 0 (0) |
| Family history of lichen sclerosus | 14 (50) | 13 (46) | 1 (4) | 0 (0) |
| Vulval hygiene |  |  |  |  |
| Washing frequency | 15 (54) | 13 (46) | 0 (0) | 0 (0) |
| Pre- prayer washing | 6 (21) | 20 (71) | 2 (7) | 0 (0) |
| Hair removal | 8 (29) | 19 (68) | 1 (4) | 0 (0) |
| Hair removal method | 9 (32) | 18 (64) | 1 (4) | 0 (0) |
| Investigations |  |  |  |  |
| Biopsy this episode | 20 (71) | 8 (29) | 0 (0) | 0 (0) |
| Patch test/ patch test results | 15 (54) | 12 (43) | 0 (0) | 1 (4) |
| Swab/ swab results | 15 (54) | 13 (46) | 0 (0) | 0 (0) |
| Medical photography | 11 (39) | 15 (54) | 2 (7) | 0 (0) |
| Urine dipstick/culture | 6 (21) | 17 (61) | 5 (18) | 0 (0) |
| Skin scraping | 1 (4) | 19 (68) | 6 (21) | 2 (7) |
| Bloods e.g. thyroid, ferritin, glucose | 4 (14) | 18 (64) | 5 (18) | 1 (4) |
| Diagnosis of vulval disease |  |  |  |  |
| **Additional diagnosis** | **22 (79)** | 6 (21) | 0 (0) | 0 (0) |
| **Previous vulval diagnoses made** | **22 (79)** | 4 (14) | 2 (7) | 0 (0) |
| Treatments/management |  |  |  |  |
| **Treatment(s) prior to referral** | **21 (75)** | 7 (7) | 0 (0) | 0 (0) |
| Date treatment started/date treatment stopped | 14 (50) | 14 (50) | 0 (0) | 0 (0) |
| Treatment regimen | 18 (64) | 10 (36) | 0 (0) | 0 (0) |
| Over the counter products used | 16 (57) | 12 (43) | 0 (0) | 0 (0) |
| Patient information given Y/N | 13 (46) | 12 (43) | 3 (11) | 0 (0) |
| Social History |  |  |  |  |
| Smoking status | 13 (46) | 12 (43) | 3 (11) | 0 (0) |
| Multidisciplinary involvement |  |  |  |  |
| Other specialties involved in care | 13 (46) | 12 (43) | 3 (11) | 0 (0) |

**Supplementary Table 3.**

Demographics related to clinical practice of Delphi survey round 1 participants:

|  | Number of participants (%) |
| --- | --- |
| Primary healthcare profession |  |
| Dermatologist | 22 (48) |
| Gynaecologist | 17 (37) |
| Sexual health practitioner | 2 (4) |
| Nurse | 2 (4) |
| Retired gynaecologist | 1 (2) |
| General Practitioner with Special Interest in Dermatology (GPSI) | 1 (2) |
| Gynae-oncologist | 1 (2) |
| Years managing patients with vulval conditions |  |
| <1 year | 1 (2) |
| 1-5 years | 8 (17) |
| 6-10 years | 11 (24) |
| 11-20 years | 16 (35) |
| >20 years | 10 (22) |
| Location of treatment provision |  |
| England | 38 (83) |
| Northern Ireland | 2 (4) |
| Scotland | 4 (9) |
| United states | 1 (2) |
| Ireland | 1 (2) |
